# Distribution pattern of black fly (Diptera: Simuliidae) assemblages along an altitudinal gradient in Peninsular Malaysia

**DOI:** 10.1186/s13071-016-1492-7

**Published:** 2016-04-19

**Authors:** Zubaidah Ya’cob, Hiroyuki Takaoka, Pairot Pramual, Van Lun Low, Mohd Sofian-Azirun

**Affiliations:** Institute of Biological Sciences, Faculty of Science, University of Malaya, 50603 Kuala Lumpur, Malaysia; Department of Biology, Faculty of Science, Mahasarakham University, Maha Sarakham, 44150 Thailand

**Keywords:** Black fly, *Simulium*, Elevation, Habitat characteristics, Peninsular Malaysia

## Abstract

**Background:**

Preimaginal black flies (Diptera: Simuliidae) are important components of the stream ecosystem. However, there has been limited research undertaken on the vertical distribution of preimaginal black flies and their associated ecological factors. Stream conditions are generally variable along the altitudinal gradient. Therefore, we conducted an in-depth entomological survey to investigate the simuliid distribution pattern along an altitudinal gradient in Peninsular Malaysia.

**Methods:**

A total of 432 collections were performed in this study (24 samplings at each of 18 fixed-streams at monthly intervals) from February 2012 to January 2014. Larvae and pupae attached on aquatic substrates such as grasses, leaves and stems, twigs, plant roots and rocks were collected by hand using fine forceps. Stream depth (m), width (m), velocity (m/s), water temperature (°C), acidity (pH), conductivity (mS/cm) and dissolved oxygen (mg/L) were measured at the time of each collection.

**Results:**

A total of 35 black fly species were recorded in the present study. The most frequently collected species were *Simulium tani* (31.7 %) and *S. whartoni* (21.5 %), while the relatively common species were *Simulium* sp. (nr. *feuerborni*) (16.2 %), *S. decuplum* (15.5 %), *S. angulistylum* (14.8 %), *S. bishopi* (13.2 %) and *S. izuae* (11.8 %). Total estimated species richness ranged between 39.8 and 41.3, which yielded more than 80 % of sampling efficiency. Six simuliid species were distributed below 500 m, whereas eight species were distributed above 1400 m. *Simulium* sp. (nr. *feuerborni*) and *S. asakoae* were found from middle to high altitudes (711–1813 m). *Simulium whartoni*, *S. brevipar* and *S. bishopi* were distributed widely from low to high altitudes (159–1813 m). Regression analysis between species richness and PCs revealed that the species richness was significantly associated with wider, deeper and faster streams at low altitude, normal water temperature (23–25 °C), low conductivity, higher discharge, more canopy cover and riparian vegetation and with larger streambed particles (*F* = 20.8, *df* = 1, 422, *P* < 0.001). Forward logistic regression indicated four species were significantly related to the stream variables (*S. whartoni*, *Simulium* sp. (nr*. feuerborni*), *S. tani* and *S. angulistylum*). Canonical correspondence analysis indicated that the temperature, stream size and discharge were the most important factors contributing to the separation of the stream sites from different altitude and hence are the predictors for the distribution of black fly species assemblages.

**Conclusions:**

This study has provided insight into the distribution pattern of preimaginal black fly assemblages along an altitudinal gradient in Peninsular Malaysia. This study could deepen our knowledge on the ecology and biology of the specialised taxa in response to environmental changes.

## Background

Species community structure and distribution vary spatially in response to a broad range of environmental factors including altitude [1, 2]. Towards high altitude, the main general changes observed involve stream size [3], stream depth [4], temperature, precipitation (i.e. snow and rain), partial pressure of atmospheric gases, atmospheric turbulence and wind speed, and radiation input, including short-wave ultraviolet radiation at different wavelengths [1, 5]. Consequently, all these changes create a barrier to species and drive to community diversification [2]. Moreover, communities appear to have been gradually decreasing in taxa richness with increasing altitude [6, 7].

Black flies (Diptera: Simuliidae) are insects of medical and veterinary significance. They are vectors of human diseases, notably human onchocerciasis or river blindness, the second ranking cause of infectious blindness [8]. In addition, black flies are also the vectors of diseases transmitted among wild animals and livestock [9, 10]. On the other hand, black fly larvae are dominant inhabitants of unpolluted streams and rivers over a wide range of altitudes [11]. They are postulated to have evolved in cool and mountainous environments [8]. The climate change during the glacial periods has driven the population expansion of simuliids along latitudinal and altitudinal gradients [8, 12, 13]. However, as our climate changes, the widespread alpine taxa in the cooler regions could be fragmented and isolated as specialised taxa or high-altitude specialists [8].

Black fly assemblage is a colony of different species occurring in similar ecological or habitat requirements. Defining this assemblage is of paramount importance in evaluating the comparative richness of populations, and the effect of isolation or fragmentation [14]. Numerous factors have been linked to black flies assemblage, these include competition [15], food availability [16], substrate type [17, 18], water current velocity [19, 20], water temperature [21, 22] and altitude [23, 24]. To date, the ecological studies of black flies have been given more attention on spatial distribution in response to habitat disturbance [25, 26], seasonal variation [21, 23] and locality richness [21, 27–30] as well as the breeding habitat preference [31]. However, there has been limited research undertaken on the vertical distribution of preimaginal black flies and their associated ecological factors. The knowledge of distribution patterns related to altitude could contribute to the understanding of the geographical distribution of many species as well as their local diversity.

We hypothesized that stream conditions vary according to the altitude, thus black fly diversity and assemblages are expected to change along the altitudinal gradient. Certain preimaginal stages of black flies would have a broad range of vertical distribution (i.e. generalist species), while others might show a specific range of distribution (i.e. specialist species). The specialised taxa may limit their distribution to certain preferred microhabitat/niche conditions [25, 31, 32]. To test this hypothesis, we made our first attempt to investigate the distribution pattern of black flies and their associated environmental factors, along an altitudinal gradient in Peninsular Malaysia.

## Methods

### Study sites

**Fig. 1:**
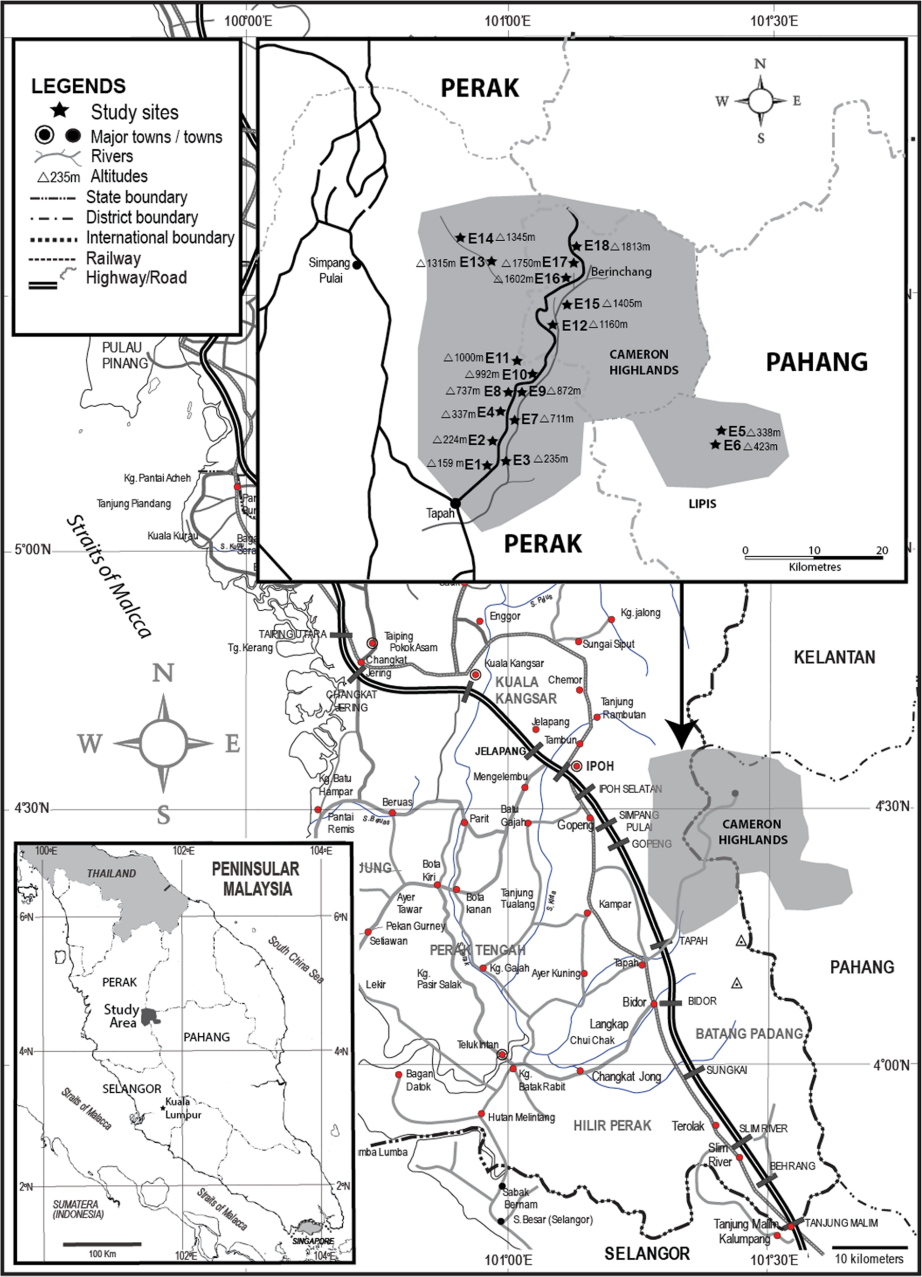
Map showing 18 fixed-streams (E1 − E18) located in the states of Pahang and Perak in Peninsular Malaysia (top right), small map showing the Peninsular Malaysia (bottom left)

A total of 18 stream points were selected as fixed sites for sampling. Streams were chosen according to their accessibility for collection, altitude and the presence of flow. These stream locations were divided into three categories: (1) low altitude (100–500 m), (2) middle altitude (501–1000 m), (3) high altitude (1001–1813 m). A total of six streams were assigned as low altitude: (E1–E6), five streams as middle altitude: (E7–E11) and seven streams as high altitude (E12–E18). Seven streams are located in the state of Pahang (Cameron Highland, 04°28.738'N, 101°22.979'E and Lipis, 04°23.715'N, 101°36.443'E) while 11 streams are located in the state of Perak (Tapah, 04°14.203'N, 101°18.354'E and Simpang Pulai, 04°34.956'N, 101°20.717'E). Location details for 18 fixed-stream sites and associated ecological characteristics are presented in Fig. 1 and Table 1.

**Table 1:** Locations of 18 fixed-streams and associated ecological characteristics

Stream/altitude	Locality	Altitude (m)	Code	GPS	Canopy cover	Riparian vegetation	Streambed
*Low*							
E1	Tapah	159	TPS1	04°14.203'N, 101°18.354'E	Partial	Forest	Rubble
E2	Tapah	224	TPS3	04°16.522'N, 101°18.996'E	Complete	Forest	Rubble
E3	Tapah	235	TPS2	04°16.316'N, 101°19.022'E	Open	Forest	Boulder
E4	Tapah	337	TPS4	04°18.420'N, 101°19.658'E	Complete	Forest	Bedrock
E5	Lipis	388	RBS18	04°23.715'N, 101°36.443'E	Open	Brush	Sand
E6	Lipis	423	RBS17	04°23.715'N, 101°36.443'E	Partial	Brush	Bedrock
*Middle*							
E7	Cameron Highland	711	CHS5	04°22.220'N, 101°21.512'E	Partial	Forest	Bedrock
E8	Cameron Highland	737	CHS6	04°22.660'N, 101°21.902'E	Complete	Forest	Small stone
E9	Cameron Highland	872	CHS7	04°23.165'N, 101°22.334'E	Open	Forest	Boulder
E10	Cameron Highland	992	CHS9	04°24.274'N, 101°22.572'E	Partial	Forest	Bedrock
E11	Cameron Highland	1000	CHS8	04°24.112'N, 101°22.356'E	Complete	Forest	Sand
*High*							
E12	Cameron Highland	1160	CHS10	04°26.723'N, 101°22.979'E	Partial	Forest	Sand
E13	Simpang Pulai	1315	SPS12	04°34.956'N, 101°20.717'E	Open	Brush	Bedrock
E14	Simpang Pulai	1345	SPS13	04°34.760'N, 101°20.507'E	Complete	Brush	Sand
E15	Cameron Highland	1405	CHT11	04°28.738'N, 101°22.979'E	Partial	Forest	Rubble
E16	Cameron Highland	1602	CHS14	04°31.258'N, 101°24.247'E	Open	Brush	Boulder
E17	Cameron Highland	1750	CHS16	04°31.519'N, 101°23.365'E	Partial	Forest	Boulder
E18	Cameron Highland	1813	CHS15	04°31.461'N, 101°23.338'E	Complete	Brush	Small stone

### Black fly sampling and identification

Overall, a total of 432 collections were performed in this study (24 at each 18 fixed-stream sites at monthly intervals) from February 2012 to January 2014. Our extensive repeated samplings were expected to reveal the most accurate species number at all surveyed streams. Thus, sampling bias due to seasonal fluctuations in physicochemical parameters was minimized in this study. Each stream was sampled from downstream to upstream (20 m), for approximately 1 h, by two people. Larvae and pupae attached on aquatic substrates such as grasses, leaves and stems, twigs, plant roots and rocks were collected by hand using fine forceps. These sampling protocols could represent the species occurrence in a locality [21, 33]. Pupae attached on similar substrates were individually kept alive in vials until emergence. The adults, together with their pupal exuviae and cocoons were preserved in 80 % ethanol for identification at the subgenus, species-group or species level. The methods of collection and identification followed those of Takaoka [34] and Adler et al. [10].

### Physicochemical measurements

The following stream physicochemical parameters were measured at the time of each collection: stream depth (m), width (m), velocity (m/s) (one to three measurements along the collection path), water temperature (°C), acidity (pH), conductivity (mS/cm) and dissolved oxygen (mg/l). The values of pH, temperature, conductivity and dissolved oxygen were taken using a portable multi probe parameter (Hanna HI 9828). Meter tape and steel ruler were used to measure stream width and depth, respectively, while a cork and a timer watch were used to measure stream velocity; the time taken for a cork to move one meter in distance. Velocity, depth and width measurements were used to estimate discharge [35]. The ecological and physicochemical measurement protocols including those for major streambed particles, riparian vegetation, and canopy cover followed those of McCreadie et al. [35]. For each fixed-stream site, the latitude and longitudinal coordinates were taken once and recorded using a hand held global positioning system (GPS) instrument (Garmin International Inc., Olathe, KS).

### Data analyses

Frequency of occurrence (FO) was designated in percentages (Table 2), calculated by the total number of a species occurrence, divided by the total number of collections (*n* = 432) [30]. Stream occurrence (SO) presented in percentages was calculated by the number of sites where a species was taken, divided by the total streams (*n* = 18). A rarefied species accumulation curve of individuals was created for all samples to determine if species in the site were adequately sampled [36]. The expected richness (First Order Jackknife and Chao estimates) was obtained to predict the possible number of species occurring in all fixed-stream sites. Species diversity estimations were calculated to determine the efficiency of the sampling by dividing the number of actual species collected by the number of estimated species [37]. To test the null hypothesis of random co-occurrence, we employed the null modeling software ECOSIM Version 7 [38] to create null models for co-occurrence, in which the C-score index [39] with fixed sums for row and column constraints was applied [40]. The presence or absence of a species was expressed on a binary scale (0 = absent, 1 = present), as in previous studies [25, 29, 41]. Cluster analysis based on Sorenson’s coefficient was used to compare the percentage of site similarity in species composition for each site. Regression analysis was used to determine relationships between species distribution and stream variables. Because stream variables are inter-correlated, principal components analysis (PCA) was used to reduce the number of variables into groups of independent components. Principal components (PCs) with eigenvalues greater than 1.0 were retained as variables. To interpret the PCs, Spearman’s rank correlation test was used to detect the relationship between principal components and stream variables using a significance level of *P* < 0.001. Forward logistic regression analysis was used to examine the relationships between spatial distribution and the PCs. Only species that occurred at more than 10 % of total collections were considered in regression analyses [30] because those present at a lower frequency have resulted in the lack of statistical power (large number of zero values were observed) [21]. Linear regression was used to test the relationship between species richness (i.e. number of species in each sampling site) and the stream variables of the sampling sites (i.e. PC scores). All collections were subjected to PCA, and the PC scores were used for regression analysis. Canonical correspondence analysis (CCA) was used to investigate the relationship between environmental variables and species assemblages. CCA was analysed using the combined data set (*n* = 432 collections). The CCA was conducted using the program PC-ORD (version 5.14) [42]. Species Diversity and Richness (SDR) version 4 [43], the SPSS statistical package, version 16.0, Chicago, IL, were employed for diversity and statistical analyses respectively.

**Table 2:** Abundance of black flies, frequency of occurrence (FO) and stream occurrence (SO) from 24 collections at 18 fixed-streams in Peninsular Malaysia

Species	Number of specimens	%	%
		FO	SO
*S*. (*Gomphostilbia*) *adleri* Jitklang & Kuvangkadilok, 2008	3	0.7	5.6
*S*. (*Gomphostilbia*) *angulistylum* Takaoka & Davies, 1995	347	14.8	55.6
*S*. (*Gomphostilbia*) *asakoae* Takaoka & Davies, 1995	837	7.6	55.6
*S*. (*Gomphostilbia*) *brinchangense* Takaoka et al., 2014	11	0.7	11.1
*S*. (*Gomphostilbia*) *burtoni* Takaoka & Davies, 1995	19	3.0	16.7
*S*. (*Gomphostilbia*) *cheongi* Takaoka & Davies, 1995	12	2.3	22.2
*S*. (*Gomphostilbia*) *decuplum* Takaoka & Davies, 1995	176	15.5	61.1
*S*. (*Gomphostilbia*) *duolongum* Takaoka & Davies, 1995	3	0.7	11.1
*S*. (*Gomphostilbia*) *gombakense* Takaoka & Davies, 1995	117	10.0	50.0
*S*. (*Gomphostilbia*) *izuae* Takaoka et al., 2013	333	11.8	55.6
*S*. (*Gomphostilbia*) *longitruncum* Takaoka & Davies, 1995	1	0.2	5.6
*S*. (*Gomphostilbia*) *lurauense* Takaoka et al., 2013	26	3.2	33.3
*S*. (*Gomphostilbia*) *roslihashimi* Takaoka & Sofian-Azirun, 2011	112	9.3	55.6
*S*. (*Gomphostilbia*) *sheilae* Takaoka & Davies, 1995	120	4.4	44.4
*S*. (*Gomphostilbia*) *sofiani* Takaoka & Hashim, 2011	31	3.2	16.7
*S*. (*Gomphostilbia*) sp. (nr. *parahiyangum*)^a^	54	6.5	38.9
*S*. (*Gomphostilbia*) *tanahrataense* Takaoka et al., 2014	1	0.2	5.6
*S*. (*Gomphostilbia*) *trangense* Jitklang et al., 2008	312	6.7	38.9
*S*. (*Gomphostilbia*) *whartoni* Takaoka & Davies, 1995	709	21.5	77.8
*S*. (*Nevermannia*) *aureohirtum* Brunetti, 1911	103	4.9	27.8
*S*. (*Nevermannia*) *caudisclerum* Takaoka & Davies, 1995	17	0.7	5.6
*S*. (*Nevermannia*) sp. (nr. *feuerborni)* ^a^	688	16.2	44.4
*S*. (*Nevermannia*) *kurtaki* Takaoka & Davies, 1995	1	0.2	5.6
*S*. (*Simulium*) *bishopi* Takaoka & Davies, 1995	305	13.2	77.8
*S*. (*Simulium*) *brevipar* Takaoka & Davies, 1995	122	10.6	77.8
*S*. (*Simulium*) *digrammicum* Edwards, 1928	1	0.2	5.6
*S*. (*Simulium*) *grossifilum* Takaoka & Davies, 1995	106	8.3	50.0
*S*. (*Simulium*) *hackeri* Edwards, 1928	96	3.7	11.1
*S*. (*Simulium*) *hirtinervis* Edwards, 1928	63	3.0	33.3
*S*. (*Simulium*) *jeffreyi* Takaoka & Davies, 1995	442	8.3	11.1
*S*. (*Simulium*) *malayense* Takaoka & Davies, 1995	188	8.6	38.9
*S*. (*Simulium*) *nobile* De Meijere, 1907	42	1.6	5.6
*S*. (*Simulium*) sp. (nr. *grisescens*)^a^	7	0.2	5.6
*S*. (*Simulium*) *tani* Takaoka & Davies, 1995	2669	31.7	66.7
*S*. (*Simulium*) *yongi* Takaoka & Davies, 1997	11	1.9	16.7

^a^Undetermined species, which probably are new species

## Results

### Black fly species composition

Thirty-five species were collected from 24 samplings at each of 18 fixed-stream sites (Table 2). The most frequently collected species (FO) were *S. tani* (31.7 %) and *S. whartoni* (21.5 %). Relatively common species were *Simulium* sp. (nr. *feuerborni*) (16.2 %), *S. decuplum* (15.5 %), *S. angulistylum* (14.8 %), *S. bishopi* (13.2 %) and *S. izuae* (11.8 %). Other species were collected at a frequency lower than 10 % and considered as rare. In terms of total individuals collected, *S. tani*, *S. asakoae*, *S. whartoni* and *Simulium* sp. (nr. *feuerborni*) were the four most abundant species. Based on stream occurrence (SO), *S. whartoni, S. bishopi* and *S. brevipar* were the widest distributed species (14 streams or 77.8 % each).

At the subgeneric level, *Gomphostilbia* was the most diverse subgenus found (19 species), followed by the *Simulium* (*s. str*.) (12 species) and the least was *Nevermannia* (four species). Of 18 species-groups in Malaysia, 17 were recorded in this study. The most abundant group was the *S. asakoae* species-group (six species), followed by the *S. epistum* species-group (four species). Other species groups were represented by one or two species.

**Fig. 2:**
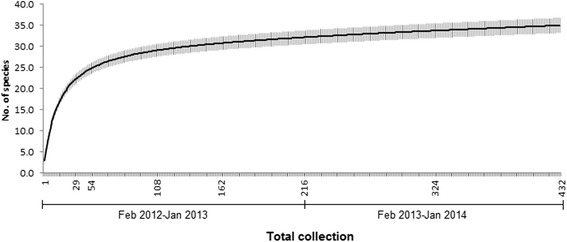
Species accumulation curve with error bars for overall 432 collections at 18 fixed-streams along an altitudinal gradient in Peninsular Malaysia

Species richness and estimated richness are presented in Table 3. The maximum number of black fly species collected per total collections was 11 and the mean number was 3.2 ± 0.1 (SE). Total estimated species richness ranged between 39.8 and 41.3, which yielded more than 80 % sampling efficiency, while the estimated species richness for each stream ranged between 4.1 and 24.0, with 60 % sampling efficiency. Species reaching asymptote after approximately 54 samplings were performed, supporting the efficiency of the sampling method used in this study (Fig. 2).

**Table 3:** Actual and estimated species richness for 18 fixed-streams along an altitudinal gradient in Peninsular Malaysia. Numbers in parentheses indicate sampling efficiency^a^

Altitudes	Actual species	Mean richness (± SE)	Chao estimates	First Order Jackknife
All	35	3.15 ± 2.00	41.3 ± 2.08 (84.7)	39.8 ± 1.95 (87.9)
Low altitude				
E1	13	2.52 ± 0.35	13.7 ± 1.10 (94.8)	16.8 ± 1.78 (77.4)
E2	15	3.43 ± 0.31	24.0 ± 7.69 (62.5)	21.7 ± 3.22 (69.1)
E3	15	3.50 ± 0.28	23.0 ± 8.31 (65.2)	19.8 ± 1.90 (75.7)
E4	8	3.21 ± 0.41	8.4 ± 0.95 (95.1)	8.95 ± 0.96 (89.4)
E5	12	4.30 ± 0.52	12.1 ± 0.92 (99.2)	12.9 ± 0.96 (93.0)
E6	14	4.64 ± 0.48	16.0 ± 3.01 (87.5)	16.9 ± 1.59 (82.8)
Middle altitude				
E7	16	5.35 ± 0.67	18.0 ± 3.01 (88.8)	17.9 ± 1.33 (89.4)
E8	12	2.33 ± 0.40	16.1 ± 11.5 (74.5)	17.7 ± 2.07 (67.8)
E9	14	2.65 ± 0.33	20.3 ± 5.89 (68.9)	18.8 ± 1.95 (74.5)
E10	5	2.67 ± 0.67	7.0 ± 3.01 (71.4)	7.8 ± 2.87 (64.1)
E11	11	3.21 ± 0.32	13.6 ± 7.78 (80.9)	13.9 ± 1.59 (79.1)
High altitude				
E12	11	3.44 ± 0.35	13.4 ± 0.60 (82.1)	12.9 ± 1.32 (85.2)
E13	16	3.34 ± 0.43	22.3 ± 5.89 (71.7)	20.8 ± 2.40 (76.9)
E14	13	3.36 ± 0.50	16.5 ± 5.66 (78.8)	17.8 ± 2.39 (73.0)
E15	13	2.83 ± 0.28	14.7 ± 6.17 (88.4)	20.7 ± 3.29 (62.8)
E16	4	1.69 ± 0.15	4.1 ± 0.37 (97.5)	4.09 ± 0.09 (97.8)
E17	7	1.29 ± 0.18	11.0 ± 1.03 (63.6)	11.2 ± 3.83 (62.5)
E18	10	1.76 ± 0.42	14.2 ± 6.82 (70.4)	16.1 ± 1.95 (62.1)

^a^Sampling efficiency was calculated by dividing the number of actual species sampled by the number of estimated species [36]

### Species diversity and distribution patterns

**Fig. 3:**
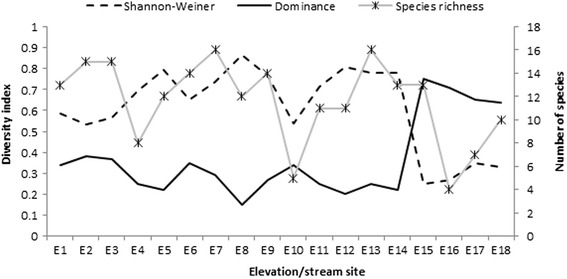
Diversity indices for black fly species along an altitudinal gradient in Peninsular Malaysia

Diversity indices are presented in Fig. 3. Species diversity and richness increased with altitude and declined at 1600 m and above. Diversity value was highest at stream E8 (0.87) followed by streams E12 (0.81), E5 (0.79) and E13 (0.78). The values were lower at streams E17 (0.35), E18 (0.33), E16 (0.27) and E15 (0.25). In contrast, dominance index (D) was highest at 1400 m and above (E15–E18).

Frequency of occurrence for distribution of 35 black fly species is presented in Table 4. Five patterns of species distribution were observed: (1) six species distributed at low altitude (159–423 m), (2) three species distributed from low to middle altitudes (159–1000 m), (3) two species distributed from middle to high altitudes (711–1813 m) (4) eight species distributed at high altitudes (1405–1813 m) and (5) 16 species distributed from low to high altitudes (Table 4).

**Table 4:** Frequency of occurrence per 24 collections for 35 black fly species along an altitudinal gradient in Peninsular Malaysia

Species	Site																	
	Low						Middle					High						
	E1	E2	E3	E4	E5	E6	E7	E8	E9	E10	E11	E12	E13	E14	E15	E16	E17	E18
*S.* sp. (nr*. grisescens*)		1																
*S. duolongum*	2	1																
*S. nobile*			7															
*S. cheongi*	2	1	3		5													
*S. adleri*						3												
*S. jeffreyi*		16	20															
*S. lurauense*		2	2		3	1	5	1										
*S. yongi*				3			2		3									
*S. sheilae*	7	2	1	1	3	2	1				2							
*S. angulistylum*	16	2	4	5	13	18	2			1	2	1						
*S*. sp. (nr*. parahiyangum*)	1	15	6		3	1			1				1					
*S. roslihashimi*	6		1		1	5	7	4	1		12	1		2				
*S. trangense*	15	4	4			3		1	1					1				
*S. grossifilum*	1	1	2	8			16	1	6				1	1				
*S. malayense*		1				5	18	6	5				1	1				
*S. izuae*	1					1	7	8	2		13	10	2	6	1			
*S. burtoni*		5	7												1			
*S. hirtinervis*		1	1				6						1		1	3		
*S. decuplum*	2		2		16	14	10		3			9	6	2	1		1	
*S. gombakense*					12	11	2	3	1	2	6	6					1	
*S. brevipar*	2			2	2	8	3	2	5	3	2	5	3	7			1	1
*S. bishopi*	1			9	9	4	11	1	1	1	1	4	8	5	1			1
*S. aureohirtum*	2												4		4	10		1
*S. tani*		22	21	14	3		21	1	11		1	4	14	5	20			1
*S. whartoni*		1	1	3	14	19	6	3	2	1	15	9	5	11	1		1	1
*S. asakoae*							1	1	1		1	3	2		1	21	2	2
*S.* sp*. (*nr*. feuerborni)*											2	6	4	4	19	6	14	15
*S. digrammicum*													1					
*S. longitruncum*														1				
*S. hackeri*													9		7			
*S. sofiani*													4	1	9			
*S. tanahrataense*															1			
*S. brinchangense*																	2	1
*S. kurtaki*																		1
*S. caudisclerum*																		3

**Fig. 4:**
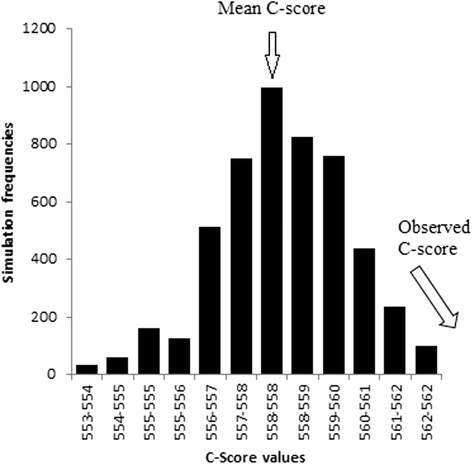
Simulated and observed values of the C-score for the co-occurrence of the simuliid species collected at 18 fixed-streams along an altitudinal gradient in Peninsular Malaysia

Of total collections (*n* = 432), 76.6 % (or 331) showed co-existence of species. Simulated and observed values of the C-score are presented in Fig. 4. The null model for co-occurrence indicates our observed index is above the simulated indices [observed index = 572.01, mean of simulated indices = 558.83, variance of simulated indices = 2.75, *P* (observed ≤ expected) = 1.00, *P* (observed ≥ expected) = 0.00], therefore, the distributional patterns of species were not considered random.

**Fig. 5:**
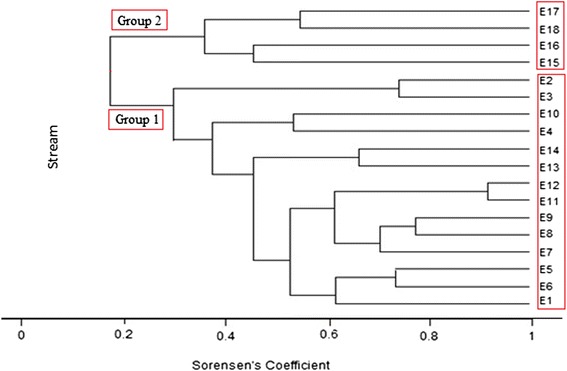
Cluster analysis based on Sorenson’s coefficient for site similarity along an altitudinal gradient in Peninsular Malaysia

Stream similarity based on species composition is presented in Fig. 5. Streams were clustered into two main groups with similarity value at 18 %: (1) ≤ 1345 m (E1–E14) and (2) ≥ 1405 m (E15–E18).

Means and coefficient of variations for eight measured physicochemical variables of all collections are presented in Table 5. PCA of all collections revealed five PCs, which have eigenvalues >1.0 accounted for 71.2 % of total inter-site variance of the physicochemical conditions (Table 6). Spearman’s rank correlations revealed that sites with higher PC-1 (which explained 24.8 % of the total variance) were at low altitude, normal water temperature (23–25 °C), wider, deeper, faster, low conductivity, higher discharge, more canopy cover and riparian vegetation and with larger streambed particles. Sites with higher PC-2 (which explained 15.1 % of the total variance) scores were at high altitude, cooler stream, wider, faster, higher dissolved oxygen with high discharge, less canopy cover and riparian vegetation. PC-3 accounted for 13.8 % of the total variance. Sites with higher PC-3 scores were at high altitude, cooler stream, smaller, slower, low conductivity and pH with low discharge and smaller streambed particles. PC-4 explained 9.3 % of the total intersite variance. Sites with higher PC-4 scores were at high altitude, cooler stream, high dissolved oxygen and pH with more canopy covers and larger streambed particles. PC-5 accounted for 8.4 % of the total variance. Streams with higher PC-5 scores were faster, high conductivity, low oxygen, more stream canopy covers and larger streambed particles. Regression analysis revealed that species richness was significantly associated with PC-1 (*F* = 20.8, *df* = 1, 422, *P* < 0.001).

**Table 5:** Physicochemical characteristics of all study sites presented as mean and coefficient of variation (CV)

Altitude/Site		Parameter	Temperature (°C)	Width (m)	Depth (m)	Velocity (m/s)	Conductivity (mS/cm)	Do (mg/l)	pH	Discharge (m^3^/s)
Low altitudes	E1	mean	25.03	0.89	0.14	0.42	0.15	4.84	6.51	0.07
		CV	4.89	106.67	47.46	14.73	19.77	76.62	10.8	157.53
	E2	mean	24.13	5.38	0.18	0.47	0.20	5.54	6.79	0.47
		CV	3.12	16.83	31.99	27.11	18.72	68.29	8.88	55.1
	E3	mean	24.26	5.7	0.16	0.44	0.18	5.67	6.93	0.41
		CV	11.24	28.31	42.37	21.51	23.96	71.67	12.39	73.48
	E4	mean	22.55	3.15	0.19	0.65	0.14	5.37	6.99	0.36
		CV	5.09	49.38	39.05	32.43	11.39	53.42	11.00	61.65
	E5	mean	23.92	0.61	0.12	0.59	0.44	5.94	6.85	0.04
		CV	4.12	30.35	34.65	21.93	16.23	33.45	9.65	52.67
	E6	mean	23.39	0.88	0.1	0.52	0.45	5.84	7.21	0.05
		CV	5.33	30.8	59.13	30.36	21.99	30.43	8.08	78.22
Middle altitudes	E7	mean	21.68	1.64	0.22	0.53	0.34	5.54	7.31	0.23
		CV	5.91	38.3	66.78	17.85	18.73	39.59	6.62	130.2
	E8	mean	21.55	0.52	0.05	0.34	0.60	5.48	7.34	0.01
		CV	4.09	38.99	29.14	23.56	13.75	38.92	7.12	53.62
	E9	mean	20.21	1.81	0.14	0.52	0.33	6.35	7.24	0.15
		CV	5.15	50.59	41.25	31.59	14.7	35.53	7.80	84.73
	E10	mean	20.42	0.93	0.14	0.38	0.25	7.52	7.52	0.07
		CV	2.98	61.12	44.8	6.00	50.00	7.63	5.13	73.4
	E11	mean	19.36	0.37	0.09	0.35	0.43	5.18	7.06	0.01
		CV	3.97	57.45	88.63	23.1	25.51	43.15	8.04	116.13
High altitudes	E12	mean	17.71	0.56	0.09	0.34	0.17	3.89	7.00	0.02
		CV	5.69	38.44	41.64	24.44	49.45	59.67	8.67	75.36
	E13	mean	18.65	3.07	0.07	0.67	0.53	6.27	7.19	0.16
		CV	6.46	49.6	103.01	23.95	24.91	29.87	12.35	134.23
	E14	mean	18.34	0.73	0.05	0.39	0.23	6.49	6.72	0.01
		CV	4.13	15.05	34.01	42.18	18.2	18.95	11.36	54.31
	E15	mean	17.22	3.17	0.22	0.54	0.18	5.94	6.79	0.37
		CV	4.93	20.22	24.45	25.36	33.85	34.93	9.76	50.13
	E16	mean	17.9	1.03	0.15	0.42	0.33	5.66	6.65	0.09
		CV	6.72	127.76	43.18	28.77	51.06	32.39	10.53	194.76
	E17	mean	17.99	0.62	0.14	0.33	0.18	5.23	6.56	0.02
		CV	6.57	37.14	174.22	36.32	33.86	42.58	9.21	117.08
	E18	mean	17.39	1.35	0.06	0.37	0.71	4.31	6.49	0.03
		CV	8.09	129.59	38.68	69.16	93.68	61.94	8.78	154.23

**Table 6:** Principal component analysis and Spearman’s rank correlation coefficients between stream variables and principal components for all collections (*n* = 432)

Variable	Stream sites			Principal components				
	Min	Max	Mean ± SE	PC-1	PC-2	PC-3	PC-4	PC-5
Altitude (m)	159.00	1813.00	891.40 ± 30.1	−0.751^**^	0.268^**^	0.522^**^	0.224^**^	0.049
Temperature (°C)	14.40	28.40	25.30 ± 0.17	0.636^**^	−0.230^**^	−0.622^**^	−0.285^**^	−0.059
Width (m)	0.12	7.90	2.95 ± 0.11	0.692^**^	0.322^**^	−0.132^**^	0.044	0.037
Depth (m)	0.02	0.85	0.54 ± 0.005	0.616^**^	0.171	0.34	0.034	0.124
Velocity (m/s)	0.16	1.03	0.42 ± 0.009	0.416^**^	0.544^**^	−0.217^**^	0.115	0.360^**^
Conductivity (mS/cm)	0.01	0.15	3.48 ± 0.001	−0.297^**^	0.162	−0.327^**^	0.001	0.328^**^
Dissolved oxygen (mg/l)	1.27	17.20	15.77 ± 0.14	0.148	0.336^**^	−0.051	0.363^**^	−0.576^**^
pH	4.32	8.53	5.20 ± 0.04	0.036	−0.080	−0.337^**^	0.739^**^	−0.155
Discharge (m^3^/s)	0.002	1.56	2.23 ± 0.01	0.786^**^	0.376^**^	−0.208^**^	0.049	0.137
Canopy cover	open	complete	2^a^	0.213^**^	−0.718^**^	0.175	0.315^**^	0.306^**^
Riparian	open	forest	2^a^	0.400^**^	−0.763^**^	0.174	0.122	−0.015
Streambed	sand	bedrock	3^a^	0.449^**^	0.176	−0.503^**^	0.274^**^	0.366^**^
% Variance explained in PCA								
Proportion				24.8	15.1	13.8	9.3	8.4
Cumulative				24.8	39.7	53.6	62.8	71.2

** *P* < 0.001

^a^Median values given for riparian vegetation (1 = open, 2 = brush and 3 = forest), streambed-particle size (min; 1 = mud/silt and max; 6 = bedrock), and canopy cover. (1 = open, 2 = partial and 3 = complete). Rankings followed McCreadie et al. [34]

Regression analysis for four black fly species is presented in Table 7. Forward logistic regression analyses were conducted for eight species, which were found in more than 10 % (FO) of total collections. All regression models of species distribution except *S. decuplum*, *S. izuae*, *S. brevipar* and *S. bishopi*, were significant at *P* < 0.001 with correct classification varying from 73.4 to 85.3 %. *Simulium whartoni* was positively associated with PC-1 and PC-3. *Simulium* sp. (nr. *feuerborni*) was positively associated with PC-3, *S. tani* was positively associated with PC-1, PC-2 and PC-4. *Simulium angulistylum* was positively associated with PC-1 but negatively associated with PC-3 and PC-4. PC-5 was not related to any species.

**Table 7:** Regression analysis for the distribution of preimaginal black fly species at 18 fixed-streams along an altitudinal gradient in Peninsular Malaysia

Species	Regression coefficient						*P*	% Correct
	*K*	PC-1	PC-2	PC-3	PC-4	PC-5		
*Simulium whartoni*	-1.048	0.403	–	0.741	–	–	< 0.001	73.4
*Simulium* sp*.*(nr. *feuerborni*)	-1.449	–	–	1.214	–	–	< 0.001	78.1
*Simulium tani*	-0.461	1.837	0.590	–	0.393	–	0.001	79.3
*Simulium angulistylum*	-2.463	0.805	–	-2.012	−0.444	–	< 0.001	85.3

**Fig. 6:**
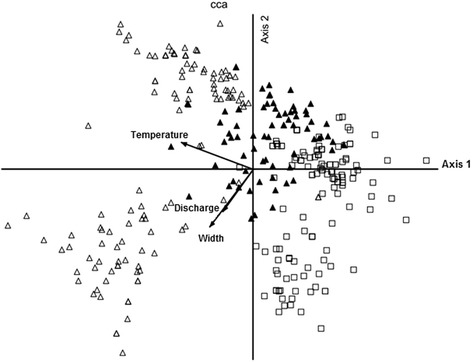
Ordination diagram of the first two axes of canonical correspondence analysis (CCA) of 432 sampling collections (open triangles represent low-altitude sites; closed triangles represent middle-altitude sites; and open square represent high-altitude sites). Arrows denote environmental variables with strength of the environmental condition indicated by arrow length of closeness to the CCA axis

**Fig. 7:**
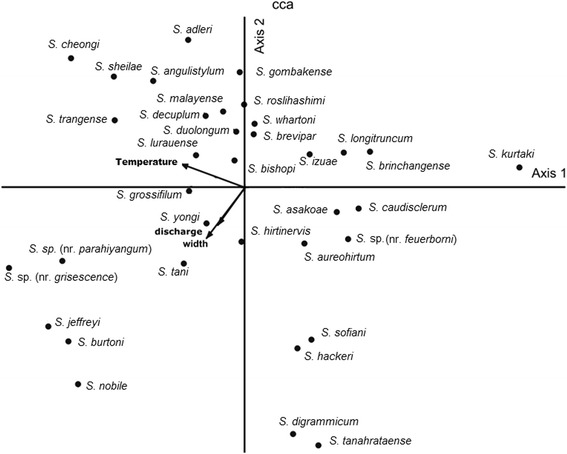
Ordination diagram of the first two axes of canonical correspondence analysis (CCA) of the 35 black fly species in Peninsular Malaysia

An ordination diagram for 18 fixed-stream sites and species are presented in Figs. 6 and 7, respectively. CCA indicated that temperature, stream size and discharge were the most important factors in differentiating streams from different altitudes. Therefore, these factors are good predictors for black fly species assemblages. The relationship between species and stream variable conditions was high (> 0.569) for the first three canonical axes, indicating that the variables used in this study were strongly related to black fly species assemblage. Temperature was the most important factor on the CCA axis 1. Species that associated with normal stream temperature were *S. cheongi* and *S. trangense*. The bottom left panel of the biplot is characterized by streams with wider and higher discharge. These sites were predominated by *S. tani*, *S. nobile* and *S. jeffreyi*. The upper right side of the biplot is composed of sites with lower discharge and smaller streams. Black fly species found predominantly at these sites were *S. bishopi*, *S. izuae* and *S. longitruncum*. The bottom right panel of the biplot is characterized by low water temperature, which is characteristic of high altitude streams. Black flies predominating at these sites were *S. asakoae*, *S. caudisclerum* and *Simulium* sp (nr. *feuerborni*) (Fig. 7).

## Discussion

This in-depth survey on the vertical distribution of black flies was conducted for the first time in Peninsular Malaysia and yielded 35 species, representing 42.7 % of total simuliids in Malaysia (82 species) [44]. *Simulium digrammicum* from Cameron Highland, an earlier described species and previously considered as locally extinct, was discovered in our study. These findings indicated that all sampled streams are the natural breeding habitats for black flies. As far as medico-veterinary importance of black flies is concerned, we collected *S*. (*G*.) *asakoae*, one of the well-known species that have been reported to be infected with filarial parasites in Thailand [45]. The host biting habits and vectorial capacity of Malaysian black flies, however, remain unknown.

Average number of species per total collections in this study was 3.2, slightly higher than previously reported [21, 25, 30–32]. Our study also revealed 74.3 % of the sampled black flies as rare species (FO < 10 %). This pattern was consistent with our previous study in Peninsular Malaysia [31] and other geographical regions [26, 30, 46]. In contrast, 22.8 % (eight species) of total species had higher frequency of occurrence (10.6 to 31.7 %), stream occurrence (44.4 to 77.8 %) and total specimens collected, confirming the previous observations where the species were relatively abundant, on average and also widely distributed [47, 48]. The capability of these species to adapt over a broad range of stream physicochemical conditions has allowed them to occur almost in all places and become generalist. In contrast, rare species require a more specialised habitat, which consequently limits their distribution to certain streams conditions and defines them as specialist. This situation also corroborated with the prediction on taxa distribution [49] and neutral theory [50].

This study showed that the five principal components had eigenvalues > 1.0 and accounted for 71.2 % of total intersite variance of the stream condition variables. Streams at low altitude, with normal water temperature (23–25 °C), wider, deeper and faster, low conductivity, higher discharge, more canopy covers and riparian vegetations and with larger stream-bed particles, accommodate more preimaginal black fly species. Some of these core factors were consistent with previous studies [21, 23, 30–32, 51, 52].

The first two CCA axes indicated the differentiation of low, middle and high altitude streams (Fig. 5). As expected, temperature, stream size and discharge are varied as altitude increases. The general conditions of low altitude streams were warmer, wider and higher discharge with average mean 23.8 °C, 2.9 m, and 0.2 m^3^/s respectively. High altitude sites were cooler (17.8 °C) than middle altitude sites (20.6 °C). However, the average mean for stream size and discharge observed both at middle (1.0 m; 0.09 m^3^/s) and high (1.5 m; 0.1 m^3^/s) altitudes were less different. In fact, stream sites at these altitudes are smaller and slower compared to low altitude sites. Our results showed gradual decrease of water temperature values as altitude increased. Dudgeon [53] suggested low water temperature as the most characteristic feature of high altitude streams. This finding is consistent with previous studies in other geographical regions [3, 4]. Besides, Tomanova et al. [4] reported stream depth as another factor that negatively related to altitude. Regarding difference in altitudes, Srisuka et al. [23] indicated that certain Thai simuliids occurred exclusively in a single zone while others were found in almost all gradients. This result was consistent with a previous study on other aquatic macroinvertebrates [3]. Our study corroborates this trend, species such as *Simulium* sp. (nr. *feuerborni*) of the subgenus *Nevermannia* and *S. asakoae* of the subgenus *Gomphostilbia* were found to be restricted to middle and high altitudes. The Thai *S. asakoae*, however, was distributed from low to high altitudes (500–2100 m) with predominance at low altitude [23]. Based on these distinct ecological conditions between Thai populations and Malaysian populations, coupled with the previous genetic evidence [54], we suggest the presence of cryptic species in *S. asakoae*. Three species in each of the subgenus *Gomphostilbia* (*S. duolongum*, *S. cheongi* and *S. adleri*) and the subgenus *Simulium* (*S. nobile*, *S. jeffreyi* and *Simulium* sp. nr. *grisescens*) were distributed at low altitude streams in our study (e.g. 159–423 m), further supporting previous published ecological data [55, 56]. Nearly half of total species collected (45.7 % or 16 species) were euryzonal or showing wide vertical distributions. These species, for example, *S. brevipar*, *S. whartoni* and *S. bishopi* were found occupying more than 80 % (16 streams) of total surveyed streams varying from low to higher altitudes. *Simulium tani* and *S. angulistylum* were found at 12 and ten altitudes, respectively (Table 4). A similar trend observed in Thai simuliid species such as *S. yuphae* were found distributed from 650–2534 m [23]. This wide vertical distribution pattern was also observed in other aquatic macroinvertebrates in temperate streams [53]. Black fly species that are widely distributed and adaptable in various physicochemical conditions are likely to be a species complex [57]. This situation has been highlighted in previous studies where the Thai *S. tani* and *S. angulistylum* were found to be a cytological species complexes [58, 59]. Our results revealed that some of the widespread species (i.e. *S. tani*, *S. angulistylum*, *S. bishopi*) along this altitudinal gradient were also commonly found in other locations in Peninsular Malaysia [31]. Cryptic diversity might be found in Malaysian samples and further cytogenetic and molecular studies would help to clarify this hypothesis.

The observed spatial distributions of preimaginal black flies in our study were predictable on the basis of stream-site characters. Our results revealed that distributions of four common species were related to altitude, temperature, stream size, velocity, streambed particle and discharge. Most of these factors are consistent with the patterns observed in tropical streams in the Oriental region and other geographical regions [21, 25, 30]. Temperature is a well-known variable that reversely correlated with altitude and has been widely associated with black fly distribution [21, 23, 25, 30, 32]. Moreover, Henriques-Oliveira & Nessimian [3] indicated that both temperature and stream size were the influencing factors in other aquatic insect distribution and composition in Southeastern Brazil. Based on our observation, species such as *Simulium* sp. (nr. *feuerborni*) was largely found in cooler streams (E11-E18) with temperature ranging between 14 °C and 19 °C and the mean between 17 °C and 19 °C. In a broader context, this species was reported as a species complex, which comprised cytoforms A and B in Thailand [60], cytoform C in Malaysia and cytoform D in Indonesia [61]. In particular, high frequency of B chromosome was detected in the *S. feuerborni* cytoform C, a unique character for temperate and arctic species [61]. Similarly, *S. caudisclerum*, S*. hackeri*, *S. digrammicum* and *S. tanahrataense* were inhabitants of cooler streams [55, 62]. The populations of these high-altitude specialists are fragmented and isolated at high altitude probably as a result of a glacial period, and thus, considered more vulnerable to extinction [8, 63]. In contrast, species such as *S. cheongi* was a common inhabitant at normal stream temperatures (23.9–25 °C) [55]. Based on our observation, the availability of larger streams with higher discharge rates gradually decreases with increasing altitude. Species such as *S. jeffreyi* and *S. nobile*, were largely associated with these stream characters and abundantly found at low altitude. In fact, stream velocity has been emphasized as one of the important factors determining the distribution of black fly larvae [21, 25, 30, 64–66].

Species richness and composition are strongly interconnected with their habitat characteristics [30]. Species richness could increase with altitude but decline at above 1500 m [2, 6, 7, 23, 67]. Our study corroborated these trends, where species richness started to decline at 1405 m. Similar results found with other macroinvertebrates where taxa richness starts to decrease as altitude increases [3, 68–70]. Regarding species composition observed in cluster analysis, there was a marked separation of streams at similar altitude (1405 m). This reflects the remarkable change of abiotic factors particularly in the observed water temperature and thus creates a boundary for most of the species with narrow ecological tolerance except several generalists and highly specialised taxa or high-altitude specialists. A similar pattern was reported in other macroinvertebrate studies along altitudinal gradients [70, 71].

Regards to species co-occurrence pattern, the assemblages observed in this study were not random and most of our samplings recorded the co-existence of species (76.6 % or 331), implying high stream environmental heterogeneity (e.g. larger stream with larger streambed particles) [25]. However, species co-existence could also be found in homogenous streams with respect to the availability of microhabitats [40]. A group of species in this particular stream would require similar microhabitat preference [40, 72]. A recent study indicated that habitat filtering is a major factor that shaping community structure of black flies in tropical streams [66]. Co-existence species usually show similar morphological traits that associate with stream conditions. Our results revealed that most of the co-occurring species possess similar labral fan morphologies, the food-filtering organ that is strongly associated with stream conditions [73]. These species for example, *S. angulistylum* and *S. trangense* were found co-existing in 50 % of total samplings, while other species such as *S. tani*, *S. jeffreyi* and *Simulium* sp (nr. *parahiyangum*) were found co-occurring in 46 % of total samplings. Therefore, patterns of species assemblage of the black flies in tropical streams in Malaysia mirror previous findings in Thailand and suggest that ecological conditions of the larval habitat play a significant role in determining black fly species assemblage.

## Conclusions

In conclusion, this comprehensive surveillance on the vertical distribution of black flies was conducted for the first time in Peninsular Malaysia and yielded 35 species, representing 42.7 % of total simuliids in Malaysia. The current study has provided new insight into the distribution patterns of preimaginal black fly along an altitudinal gradient in Peninsular Malaysia. Our results indicated that physicochemical characteristics of the stream habitats that are associated with black fly distribution (e.g. stream size, velocity and temperature) varied along an altitudinal gradient. Thus, species diversity and assemblages varied accordingly. We found that certain black fly species are habitat specialists, whereas some are habitat generalists and distributed in wide range of ecological conditions. These species are likely to contain cryptic taxa and further taxonomic study using cytogenetic and molecular methods are required to support this hypothesis. Moreover, this study could deepen our knowledge on the ecology and biology of the specialised taxa in response to environmental changes.
